# Deep Sternal Wound Infection Following OPCAB: Delving Deeper into the Predisposition!

**DOI:** 10.21470/1678-9741-2021-0547

**Published:** 2023

**Authors:** Jes Jose, Rohan Magoon, Jasvinder Kaur Kohli, Ramesh Kashav

**Affiliations:** 1 Department of Cardiac Anesthesiology, Sri Jayadeva Institute of Cardiovascular Sciences and Research, Bengaluru, India; 2 Department of Cardiac Anaesthesia, Atal Bihari Vajpayee Institute of Medical Sciences (ABVIMS) and Dr. Ram Manohar Lohia Hospital, Baba Kharak Singh Marg, New Delhi, India.

Dear Editor,

Predisposition to deep sternal wound infection (DSWI) following off-pump coronary artery bypass (OPCAB) grafting surgery classifies as an area of particular research interest. Given the fact that a sound evaluation of the risk factors for DSWI mandates a comprehensive approach, we wish to highlight a few important concerns pertaining to the Enginoev et al.^[1]^ study recently published in the *Brazilian Journal of Cardiovascular Surgery*.

Interestingly, the index analysis does not outline diabetes mellitus as a preoperative risk factor for DSWI (30.2% in DSWI and 26.2% in non DSWI group, *P*=0.5)^[1]^ albeit the lack of data on perioperative glycaemic control deserves attention. Appropriate to the context, the Mayo Clinic research group delineate as high as 30% increase in adverse outcomes, including infective complications for every 20 mg/dL rise in mean intraoperative glucose levels^[2]^. Moreover, specific literature linking glycaemic fluctuations with infective complications continues to accumulate over the past decade^[3,4]^. Järvelä et al.^[3]^ found a significantly heightened rate of postoperative infections in their cardiac surgical cohort manifesting repeated hyperglycaemia (39.7% incidence) as opposed to normoglycaemic or those with single hyperglycaemic episode (12.1% and 8.2%, respectively, *P*=0.019).

Furnary et al.^[4]^ reveal the independent DSWI predictive ability of post-cardiac surgery hyperglycaemia in the Portland Diabetic Project, wherein the subset with 48-hour mean blood glucose levels >200 mg/dL demonstrated a 2.2 times elevated risk of DSWI. Concomitantly, there is convincing evidence to suggest that perioperative glucose control with insulin infusion management protocols considerably attenuate the DSWI incidence^[4-6]^. Alongside the absence of perioperative glucose data, Enginoev et al.^[1]^ fail to describe the glucose management regime employed in their retrospective study^[1]^.

In addition, the authors could have elaborated whether or not any of the study participants were receiving preoperative corticosteroids^[1,7]^. Herein, a comparative account of the preoperative leucocytic counts of the DSWI and non-DSWI groups could also have added incremental value^[1,8]^. As much as we laud the endeavours of Enginoev et al.^[1]^, the points of perioperative relevance elucidated by us and the authors’ explanation would probably assist readers to comprehend this dynamic research area in a more holistic manner.

## References

[1] Enginoev S, Rad AA, Ekimov S, Kondrat’ev D, Magomedov G (2021). Risk Factors for Deep Sternal Wound Infection after Off-Pump
Coronary Artery Bypass Grafting: a Case-Control Study [ahead of
print]. Braz J Cardiovasc Surg.

[2] Gandhi GY, Nuttall GA, Abel MD, Mullany CJ, Schaff HV (2005). Intraoperative hyperglycemia and perioperative outcomes in
cardiac surgery patients. Mayo Clin Proc.

[3] Järvelä KM, Khan NK, Loisa EL, Sutinen JA, Laurikka JO, Khan JA. (2018). Hyperglycemic Episodes Are Associated With Postoperative
Infections After Cardiac Surgery. Scand J Surg.

[4] Furnary AP, Wu Y, Bookin SO (2004). Effect of hyperglycemia and continuous intravenous insulin
infusions on outcomes of cardiac surgical procedures: the Portland Diabetic
Project. Endocr Pract.

[5] Kramer R, Groom R, Weldner D, Gallant P, Heyl B (2008). Glycemic control and reduction of deep sternal wound infection
rates: a multidisciplinary approach. Arch Surg.

[6] Cotogni P, Barbero C, Rinaldi M (2015). Deep sternal wound infection after cardiac surgery: Evidences and
controversies. World J Crit Care Med.

[7] Magoon R, Choudhury A, Sahoo S, Malik V (2019). Steroids for adult cardiac surgery: The debate echoes
on. J Anaesthesiol Clin Pharmacol.

[8] Dey S, Kashav R, Kohli JK, Magoon R, ItiShri (2021). Systemic Immune-Inflammation Index Predicts Poor Outcome After
Elective Off-Pump CABG: A Retrospective, Single-Center Study. J Cardiothorac Vasc Anesth.

